# Parapapillary retinal hemangioblastoma presenting as an epiretinal membrane in a patient with Von Hippel–Lindau disease: a case report

**DOI:** 10.3389/fmed.2026.1826296

**Published:** 2026-05-01

**Authors:** Licong Liang, You Li, Xiaoshuang Jiang, Fang Lu

**Affiliations:** Department of Ophthalmology, West China Hospital of Sichuan University, Chengdu, China

**Keywords:** epiretinal membrane, histopathology, retinal hemangioblastoma, vitreoretinal surgery, Von Hippel–Lindau disease

## Abstract

**Background:**

Retinal hemangioblastoma (RH) is a benign, highly vascularized tumor arising from the neurosensory retina. It may occur sporadically or as a manifestation of Von Hippel–Lindau (VHL) disease. Here, we describe a case of parapapillary RH in a patient with VHL disease that presented clinically as an epiretinal membrane overlying the optic disc.

**Case description:**

A 6-year-old boy presented with an abnormality detected on left fundus examination. Fundus photography and optical coherence tomography revealed an epiretinal membrane on the temporal side of the optic disc causing retinal traction and deformation. Fundus fluorescein angiography demonstrated early hyperfluorescence within the lesion. Vitreoretinal surgery was performed with satisfactory visual recovery. Histopathological and immunohistochemical findings confirmed the diagnosis of RH. Genetic analysis identified a heterozygous splice-site mutation (c.464-2A>G) in the *VHL* gene.

**Conclusion:**

Parapapillary RH can masquerade as an epiretinal membrane over the optic disc, posing a diagnostic challenge. Histopathological confirmation and genetic evaluation are essential to establish the diagnosis and to identify underlying VHL disease.

## Introduction

Retinal hemangioblastoma (RH) is a benign, highly vascularized tumor arising from the neurosensory retina (1). It may occur sporadically or in association with Von Hippel–Lindau (VHL) disease, an autosomal dominant hereditary tumor syndrome caused by mutations in the *VHL* gene (2). Ocular involvement is a common manifestation of VHL disease, and RHs may develop in the peripheral retina or in the parapapillary region (1). Early recognition of these lesions is important because they may represent the initial manifestation of systemic disease (3, 4).

Parapapillary RHs are relatively uncommon and may present with variable clinical features depending on their location and growth pattern (5). In rare cases, these lesions may mimic other vitreoretinal interface abnormalities, posing diagnostic challenges (6, 7). Here, we report a pediatric case of parapapillary RH in a patient with VHL disease that clinically presented as an epiretinal membrane overlying the optic disc and was confirmed by histopathological and genetic analyses.

## Case presentation

A 6-year-old boy was referred to West China Hospital after an abnormality in the fundus of his left eye was detected during a routine physical examination one month earlier. Initial ophthalmologic examination revealed a best-corrected visual acuity of 40/200 in the left eye. No abnormalities were observed in the anterior segments of either eye or in the right fundus.

**Figure 1:**
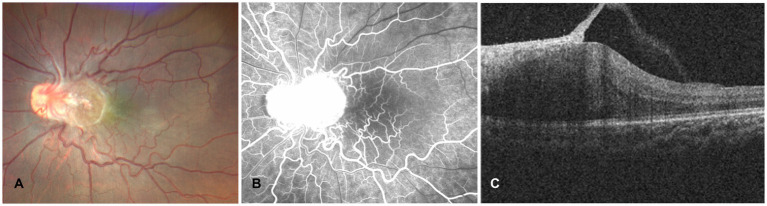
Multimodal imaging of a parapapillary RH. **(A)** Fundus photograph showing an epiretinal vascular membrane approximately 1 PD located temporal to the optic disc, with tortuous and distorted retinal vessels. **(B)** Fundus fluorescein angiography demonstrating early hyperfluorescence of the lesion. **(C)** OCT showing a hyperreflective preretinal lesion with traction on the retina.

Fundus photography (CLARUS 500, Carl Zeiss Meditec, United States) revealed an approximately 1 disc-diameter epiretinal membrane located on the temporal side of the optic disc, accompanied by tortuous and dilated retinal vessels (Figure 1). Fundus fluorescein angiography (Spectralis HRA, Heidelberg Engineering, Germany) demonstrated early hyperfluorescence within the fibrovascular membrane. Optical coherence tomography (Rescan 700, Carl Zeiss Meditec, United States) showed a localized retinal bulge associated with an epiretinal membrane.

**Figure 2:**
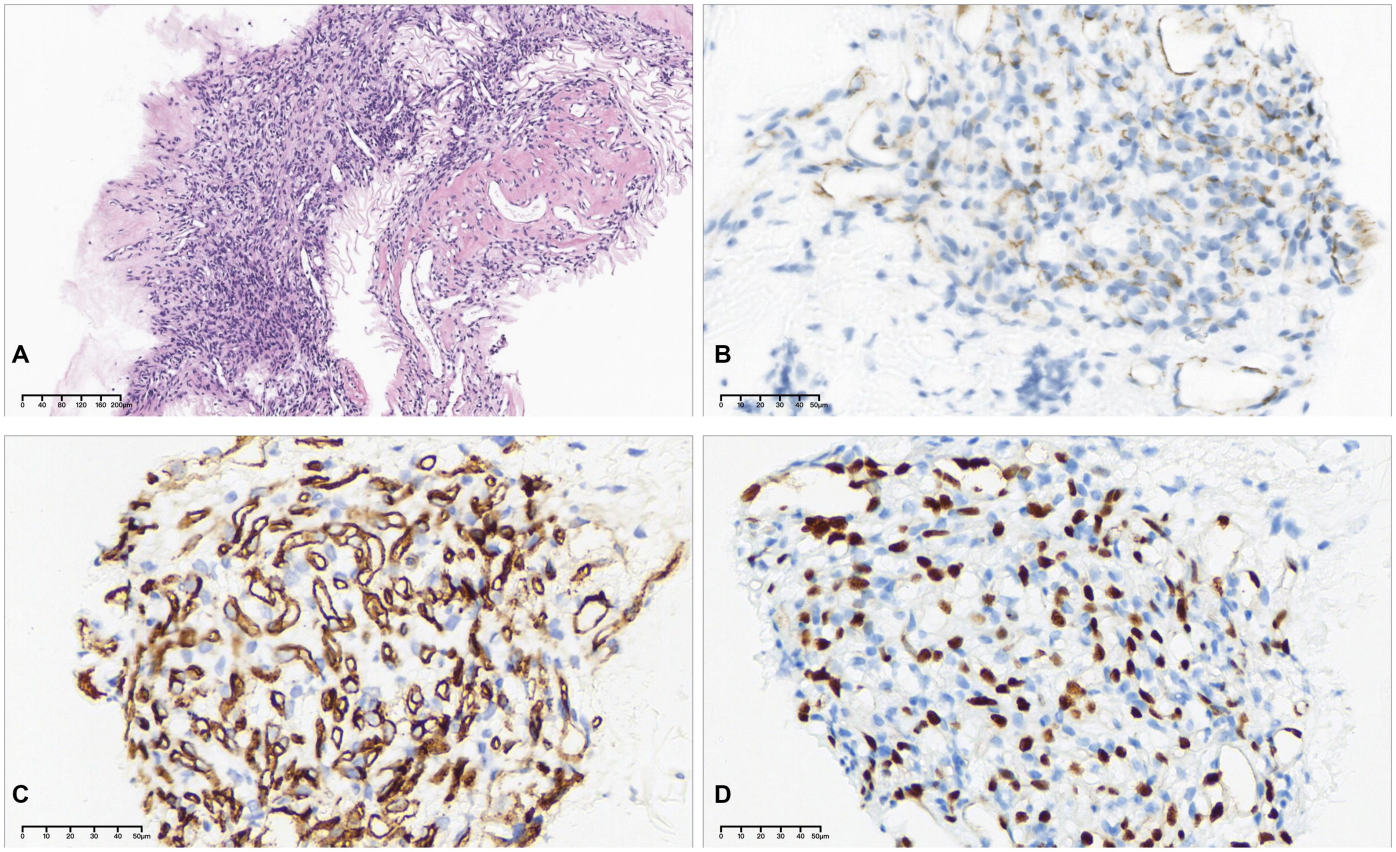
Histopathologic diagnosis of RH. **(A)** Hematoxylin and eosin (H&E) staining showing a soft tissue tumor rich in small blood vessels, with short spindle-shaped and oval stromal cells around the vessels (scale bar = 200 μm). **(B)** Immunohistochemistry for CD31 (scale bar = 50 μm). **(C)** Immunohistochemistry for CD34 (scale bar = 50 μm). **(D)** Immunohistochemistry for ERG (scale bar = 50 μm).

The patient subsequently underwent 25-gauge pars plana vitrectomy with removal of the epiretinal membrane in the left eye (Supplementary Video 1). The excised fibrovascular tissue was submitted for histopathological examination. Microscopic evaluation revealed a soft-tissue tumor composed of short spindle-shaped stromal cells and numerous small blood vessels surrounded by oval cells. Immunohistochemical staining showed positivity for CD31, CD34, ERG, inhibin (focal positive), and VHL (focal positive) (Figure 2). These histopathological and immunohistochemical findings were consistent with RH.

Genetic testing was subsequently performed for the patient and his parents. The results identified a heterozygous splice-site mutation (c.464-2A>G) in the *VHL* gene in both the patient and his mother, which was considered likely pathogenic. Based on these findings, the patient was diagnosed with VHL disease. Following the diagnosis, a comprehensive systemic evaluation was performed, including contrast-enhanced magnetic resonance imaging of the brain and orbits, as well as ultrasonography of the kidneys and urinary system, all of which revealed no additional VHL-associated lesions. The ocular manifestation was therefore considered the initial presentation of the disease.

**Figure 3:**
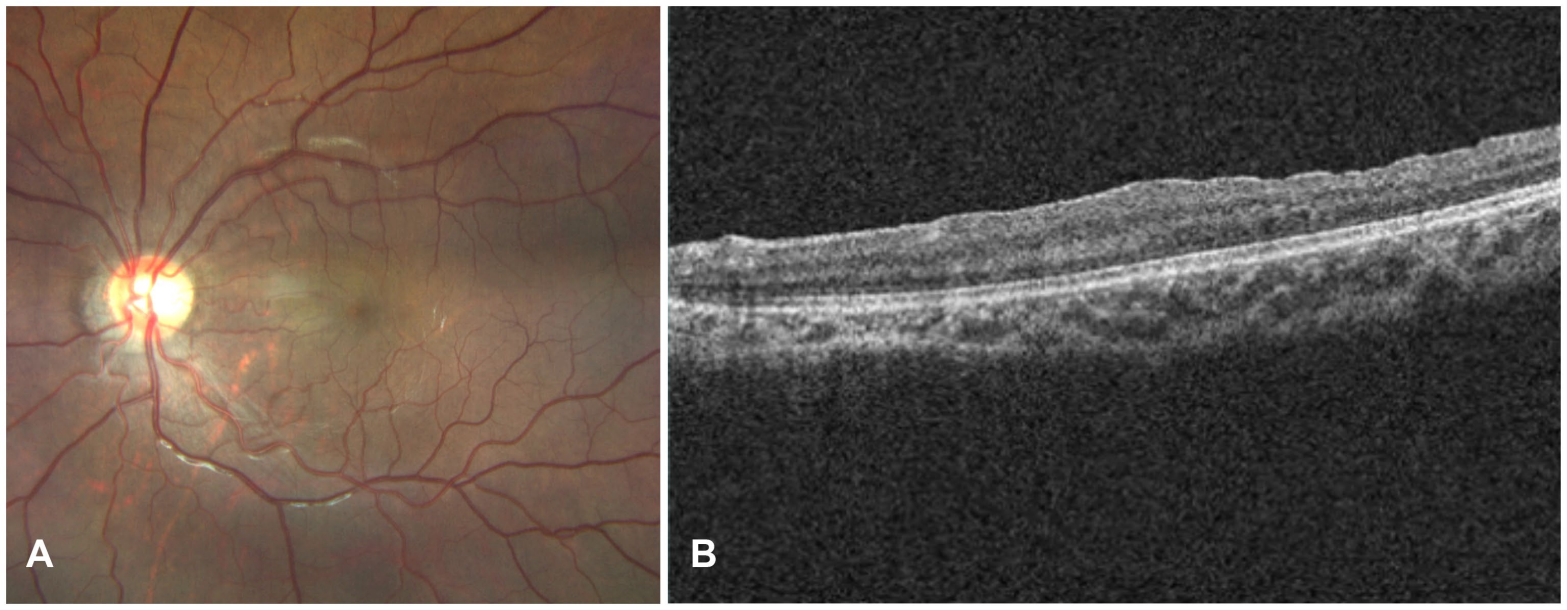
Two-year postoperative follow-up. **(A)** Fundus photograph and **(B)** OCT demonstrate complete resolution of the parapapillary hemangioblastoma, with near-normal retinal morphology restored.

At the two-year postoperative follow-up, the patient’s best-corrected visual acuity improved to 20/20 (+2.75 DS/−2.00 DC × 5). Fundus photography and OCT demonstrated resolution of the epiretinal membrane and a reduction in the bulging at the original lesion site (Figure 3). During the follow-up period, no new systemic manifestations related to VHL disease were detected.

## Discussion

RH is a benign vascular tumor of the neurosensory retina and represents one of the most characteristic ocular manifestations of VHL disease (1). Approximately 49–80% of patients with RH are associated with VHL, an autosomal dominant tumor syndrome caused by germline mutations in the *VHL* gene (2). Early recognition of RH is clinically important because it may be the first sign of underlying VHL disease, which is associated with potentially life-threatening systemic tumors including central nervous system hemangioblastomas, renal cell carcinoma, and pheochromocytoma (3).

Parapapillary RH is relatively uncommon and may present with variable clinical manifestations depending on its size and location (3, 5, 8). Typical RHs appear as reddish nodular lesions with dilated feeding and draining vessels (9). However, lesions located near the optic disc may exhibit atypical features and may be difficult to distinguish from other vitreoretinal interface abnormalities (10, 11). In the present case, the lesion presented clinically as an epiretinal membrane overlying the optic disc, producing retinal traction and distortion. This unusual appearance likely reflects the tumor’s superficial location and its interaction with the vitreoretinal interface, which can induce secondary membrane formation and tractional changes.

In the initial diagnostic evaluation, several differential diagnoses were considered, including astrocytic hamartoma, combined hamartoma of the retina and retinal pigment epithelium, neurofibromatosis type 2 (NF2)-associated epiretinal membrane, and other vitreoretinal interface abnormalities in the peripapillary region. Astrocytic hamartoma was considered unlikely, as OCT did not demonstrate the characteristic “moth-eaten” hyporeflective intralesional spaces typically observed in such lesions; instead, the lesion presented as a preretinal membrane overlying the optic disc (12). CHRRPE was also excluded based on the absence of full-thickness retinal involvement and the lack of the typical “omega (Ω) sign” on OCT (13). In addition, fundus photography did not show the characteristic grayish discoloration, and fluorescein angiography did not reveal the masking or blocked fluorescence commonly associated with CHRRPE but rather demonstrated early hyperfluorescence (14). NF2-associated epiretinal membrane was considered in the differential diagnosis, as these lesions can occur in young patients and involve the vitreoretinal interface. However, such membranes typically exhibit a characteristic “flame-like” configuration with curled edges on OCT and are generally avascular, which was inconsistent with the vascular features observed in our case (15). Given the atypical multimodal imaging findings, an NF2-related lesion was initially suspected; therefore, genetic testing was performed to confirm or exclude an underlying hereditary disorder.

The atypical presentation in our patient posed a diagnostic challenge and highlights the importance of multimodal imaging. Fundus fluorescein angiography demonstrated early hyperfluorescence within the lesion, suggesting its vascular nature (11). Genetic analysis further revealed a heterozygous splice-site mutation (c.464-2A>G) in the *VHL* gene, confirming the association with VHL disease. The identified splice-site variant affects the canonical splice acceptor site and is classified as pathogenic according to ACMG guidelines (PVS1 + PS4_Moderate + PM2_Supporting). This mutation is predicted to disrupt normal RNA splicing, likely resulting in exon skipping and consequent loss of functional pVHL protein, ultimately leading to dysregulation of hypoxia-inducible pathways. Identification of such mutations is important not only for confirming the diagnosis but also for guiding systemic evaluation and genetic counseling.

Histopathologically, RH is composed of thin-walled capillaries interspersed with polygonal or spindle-shaped stromal cells. In this case, endothelial markers (CD31, CD34, ERG) highlighted the vascular components, while stromal cells were inhibin-positive, consistent with previously reported histopathologic features, and the vascular nature was further confirmed in vitreoretinal surgical specimens (7, 16). Given the limited detailed characterization of VHL-associated RHs, especially in atypical presentations, comprehensive histopathologic and immunohistochemical evaluation remains essential for accurate diagnosis.

The management of epiretinal membranes associated with VHL disease has not been well established. Unlike in our case, where the membrane itself represented the tumor, previously reported cases have generally described these lesions as fibrocellular proliferation at the vitreoretinal interface (7, 17, 18). In such situations, surgical intervention is typically reserved for cases with significant traction or visual impairment. Previous reports indicate that some patients undergo surgical intervention at the time of ERM detection, whereas others defer PPV until systemic conditions have stabilized (6, 7). In our case, the ocular lesion represented the initial manifestation and the patient’s systemic condition was stable; therefore, surgery was performed promptly after detection. This approach not only alleviates tractional changes but also allows for definitive histopathologic diagnosis.

## Conclusion

This case emphasizes that parapapillary RH may clinically mimic an epiretinal membrane and lead to diagnostic confusion. Awareness of this atypical manifestation is important for ophthalmologists, particularly when evaluating pediatric patients with unusual peripapillary membranes. Careful fundus examination, multimodal imaging, and consideration of underlying VHL disease are essential for accurate diagnosis and appropriate management.

## Data Availability

The original contributions presented in the study are included in the article/Supplementary material, further inquiries can be directed to the corresponding author.
